# Organizational science and cybersecurity: abundant opportunities for research at the interface

**DOI:** 10.1007/s10869-021-09732-9

**Published:** 2021-02-04

**Authors:** Reeshad S. Dalal, David J. Howard, Rebecca J. Bennett, Clay Posey, Stephen J. Zaccaro, Bradley J. Brummel

**Affiliations:** 1 Department of Psychology, George Mason University, 4400 University Drive, MSN 3F5, Fairfax, VA 22030-4444 USA; 2 Department of Psychology and Muma College of Business, University of South Florida, Tampa, FL USA; 3 Department of Management, College of Business, University of Central Florida, Orlando, FL USA; 4 Cybersecurity and Privacy Cluster, University of Central Florida, Orlando, FL USA; 5 Department of Psychology, The University of Tulsa, Tulsa, OK USA

**Keywords:** Cybersecurity, Information security, Insider threat, Phishing, Social engineering, Incident response, Key performance indicators, Security operations center, Security information and event management, Multiteam system

## Abstract

Cybersecurity is an ever-present problem for organizations, but organizational science has barely begun to enter the arena of cybersecurity research. As a result, the “human factor” in cybersecurity research is much less studied than its technological counterpart. The current manuscript serves as an introduction and invitation to cybersecurity research by organizational scientists. We define cybersecurity, provide definitions of key cybersecurity constructs relevant to employee behavior, illuminate the unique opportunities available to organizational scientists in the cybersecurity arena (e.g., publication venues that reach new audiences, novel sources of external funding), and provide overall conceptual frameworks of the antecedents of employees’ cybersecurity behavior. In so doing, we emphasize both end-users of cybersecurity in organizations and employees focused specifically on cybersecurity work. We provide an expansive agenda for future organizational science research on cybersecurity—and we describe the benefits such research can provide not only to cybersecurity but also to basic research in organizational science itself. We end by providing a list of potential objections to the proposed research along with our responses to these objections. It is our hope that the current manuscript will catalyze research at the interface of organizational science and cybersecurity.

If you are reading this manuscript, you have almost certainly been the victim of a cyber data breach. No sooner have you figured out how to acquire your free credit monitoring after the Equifax data breach than you learn that Capital One Bank’s data have been accessed by an intruder. Financial agencies and credit card companies are frequent targets of intruders because of the nature of the personal data collected by the organizations. However, data breaches are by no means limited to the financial services sector: for instance, a review of breaches that occurred in 2019 conducted by Norton Internet Security (Porter, 2019) included those affecting the entertainment sector (e.g., Evite), the food delivery sector (e.g., DoorDash), the healthcare industry (e.g., American Medical Collection Agency, Zoll Medical), educational institutions (e.g., Georgia Tech), and government agencies (e.g., the Federal Emergency Management Agency). Data breaches, in other words, are prevalent across a wide spectrum of organizations.

Data breaches are also not limited by the size of the organization. A recent Data Breach Investigations Report notes that 43% of targeted attacks were directed at small businesses (Verizon, 2019) and a recent Security Threat Report notes that “employees of small organizations were more likely to be hit by email threats – including spam, phishing, and email malware – than those in large organizations” (Symantec, 2019, p. 25). Data breaches are not limited by geography either. Although the Office of Personnel Management data breach that exposed the personally identifiable information of over 20 million individuals may have dominated media headlines in the United States in 2015, no geographical location is immune to a cybersecurity breach. Recent global events include the 2019 attack on Cebuana Lhuillier, which affected 900,000 customers of the Philippines-based organization (Merez, 2019), and the 2018 attack on SingHealth, which left 1.5 million Singaporean patients (approximately 25% of the country’s population) with their personal health information compromised (Vincent, 2018).

Indeed, most organizations possess sensitive customer information (e.g., medical records, educational records, payment card data, personally identifiable information, and purchasing patterns) as well as corporate intellectual property (Posey, Raja, Crossler, & Burns, 2017). Although cybersecurity is an issue affecting virtually all organizations and their employees, the overwhelming majority of published cybersecurity research currently originates not from peer-reviewed organizational science journal articles but rather from mass media articles, corporate technical reports, and peer-reviewed journal articles from the disciplines of computer science, information systems, and information technology (e.g., Porter, 2019; Verizon, 2019). Cybersecurity attacks and breach prevention do have obvious connections with more technology-oriented disciplines, but technical expertise is not the only commodity that can aid in understanding and ameliorating cyberattacks. As a recent CNN article notes, “hackers” do not rely solely on computers to infiltrate organizational computer networks (O’Sullivan, 2019). Rather, attackers often use “social engineering” tactics to gain access to organizational networks and information they would otherwise be unable to obtain. Social engineering refers to the use of deception, manipulation, and persuasion by an attacker to attain unauthorized information from another person (Krombholz, Hobel, Huber, & Weippl, 2015; see also Table 1). Thus, cyber breaches often occur as a direct result of employees’ susceptibility to these types of attacks (e.g., being deceived into giving information) and employees’ errors and mistakes (Im & Baskerville, 2005), in addition to employees’ malicious and non-malicious noncompliance with policy (Willison & Warkentin, 2013).

**Table 1:** Cybersecurity Terms Relevant to Organizational Scientists

Cybersecurity Term	Definition/Explanation
Adversary	“An individual, group, organization, or government that conducts or has the intent to conduct detrimental activities” (NICCS, 2018). Related terms are “attacker” and “threat agent.” An “advanced persistent threat” is “[a]n adversary that possesses sophisticated levels of expertise and significant resources which allow it to create opportunities to achieve its objectives by using multiple attack vectors (e.g., cyber, physical, and deception)” (NICCS, 2018).
Behavior monitoring	“Observing activities of users, information systems, and processes and measuring the activities against organizational policies and rule[s], baselines of normal activity, thresholds, and trends” (NICCS, 2018). In organizational settings, behavior monitoring could be effected through a variety of approaches such as electronic performance monitoring (Bhave, 2014), the physical layout of workspaces (e.g., the “panopticon”; Bernstein, 2017; Bhave et al., 2020), and so forth.
Behavioral cybersecurity	“[T]he use of psychological, social, cognitive, and emotional factors as data to better understand, protect, and defend information and communication systems from any unauthorized doings, or encourage legitimate users to take better precautions” (Patterson, Winston, & Fleming, 2016, p. 257–8).
Chief Information Security Officer(CISO)	“[A] senior-level executive responsible for developing and implementing an information security program, which includes procedures and policies designed to protect enterprise communications, systems and assets from both internal and external threats. The CISO may also work alongside the [Chief Information Officer (CIO)] to procure cybersecurity products and services and to manage disaster recovery and business continuity plans” (Rouse, 2016).
[Color] hat	The color of the (figurative) hat refers to the type of hacker. “Black hat” hackers hack exploitatively whereas “white hat” hackers (also known as “ethical hackers”) hack protectively (and may, for example, be employed by organizations to assess vulnerabilities or participate in penetration tests; O’Brien & Marakas, 2011). The terms are believed to have originated from the Western genre of American movies, in which the color of the cowboy hat denoted whether a character was a villain (black hat) or a hero (white hat). Some classifications include additional colors (e.g., gray). The heart of the distinctions between the various hat colors involves motivation (e.g., black hat hackers are ostensibly motivated by personal gain or maliciousness whereas white hat hackers are ostensibly motivated by ethics/morality and law and order)—and motivation is of course a core research topic in organizational science.
[Color] team	During a cybersecurity operational exercise aimed at testing the organization’s information systems, the “red team” serves as the mock attacker, the “blue team” serves as the mock defender, and the “white team” serves to establish and monitor the rules under which the exercise is conducted (NICCS, 2018). In organizational science parlance, such operational exercises may be thought of as gamified team training exercises involving two teams of employees acting in opposition to each other.
Cyber attack	An “actual assault perpetrated by an intentional threat source that attempts to” either “alter a system, its resources, its data, or its operations” (viz., an “active attack”) or “learn or make use of information from a system, but does not attempt to alter the system, its resources, its data, or its operations” (viz., a “passive attack”) (NICCS, 2018). Various types of cyber attacks include, but are not limited to, control system attacks, phishing attacks (including the personalized spear phishing attacks), ransomware, spyware, Trojan horses, unauthorized access, viruses, and worms (Srinivas, Das, & Kumar, 2019).
Cyberdeviance	“[T]he intentional use of information technology (IT) in the workplace that is contrary to the explicit and implicit norms of the organization and that threatens the well-being of the organization and/or its members” (Venkatraman et al., 2018, p. 1061).
Cyber-physical systems	“[S]ystems which offer integrations of computation, networking, and physical processes” (Khaitan & McCalley, 2014, p. 350); for instance, the Internet of Things. The rise of cyber-physical systems is aiding in the convergence of cybersecurity and traditional physical security.
Cybersecurity	“Strategy, policy, and standards regarding the security of and operations in cyberspace, and encompass[ing] the full range of threat reduction, vulnerability reduction, deterrence, international engagement, incident response, resiliency, and recovery policies and activities, including computer network operations, information assurance, law enforcement, diplomacy, military, and intelligence missions as they relate to the security and stability of the global information and communications infrastructure” (NICCS, 2018). See also “organizational cybersecurity.”
Cybersecurity incident	“An occurrence that actually or potentially results in adverse consequences to (adverse effects on) (poses a threat to) an information system or the information that the system processes, stores, or transmits and that may require a response action to mitigate the consequences” (NICCS, 2018).
Cybersecurity Incident ResponseTeam (CSIRT)	“Group of individuals usually consisting of Security Analysts organized to develop, recommend, and coordinate immediate mitigation actions for containment, eradication, and recovery resulting from computer security incidents” (SEI, 2014). Also referred to as a Computer Emergency Response Team, Computer Security Incident Response Team, Computer Incident Response Center, Computer Incident Response Capability, or Cyber Incident Response Team (SEI, 2014). See also: Security Operations Center (SOC).
Cyberspace	“The interdependent network of information technology infrastructures, that includes the Internet, telecommunications networks, computer systems, and embedded processors and controllers” (NICCS, 2018).
Disgruntled employee	“An insider who is upset with the organization and desires to get back at it (e.g., the victim organization rejected a contract for the insider’s own firm, and the insider plots to make the new systems administrator look bad)” (Costa, Albrethsen, Collins, Perl, Silowash, & Spooner, 2016, p. 70). In organizational science parlance, this is an employee with low perceptions of organizational justice and high job dissatisfaction.
End-user	The person for whom products or policies are designed. In an organizational cybersecurity context, the end-user would be any employee who interacts with the organization’s information communication technology. The end-user is unlikely to possess the technical expertise necessary to fully understand the technology he or she is using (Cybersecurity glossary, n.d.).
Enterprise Security Risk Management	“The application of fundamental risk principles to manage all security risks—whether related to information, cyber, physical security, asset management, or business continuity—in a comprehensive, holistic, all-encompassing approach” (Allen & Loyear, 2017, p. 4).
Event	“An observable occurrence in an information system or network” (NICCS, 2018). An event “[s]ometimes provides an indication that an incident is occurring or at least raises the suspicion that an incident may be occurring” (NICCS, 2018). Contrast with: “incident.”
Hacker	“An unauthorized user who attempts to or gains access to an information system” (NICCS, 2018).
Honeypot	“[A] realistic but dummy [system]…designed to attract malicious users to inappropriately access resources…. [A honeypot provides] a mechanism to determine what motivates the inside attacker and what capabilities the attacker possesses” (Maybury et al., 2005).
Hot wash	“[A] debrief conducted immediately after an exercise or test with the staff and participants” (Cybersecurity glossary, n.d.); an after-action review.
Incident	“An occurrence that constitutes a violation or imminent threat of violation of security policies, security procedures, or acceptable use policies” (NICCS, 2018). Contrast with: “event.”
Incident response	“[C]ybersecurity work where a person [or team]: Responds to crisis or urgent situations within the pertinent domain to mitigate immediate and potential threats; uses mitigation, preparedness, and response and recovery approaches, as needed, to maximize survival of life, preservation of property, and information security” (NICCS, 2018).
Insider threat	“A person or group of persons within an organization who pose a potential risk through violating security policies” (NICCS, 2018). In organizational science parlance, this refers to an employee who poses a potential risk of engaging in cybersecurity-related counterproductive work behavior. Insider threat is contrasted with “outsider threat”: “A person or group of persons external to an organization who are not authorized to access its assets and pose a potential risk to the organization and its assets” (NICCS, 2018).
Organizational cybersecurity	The efforts organizations take to protect and defend their information assets, regardless of the form in which those assets exist, from threats internal and external to the organization. See also “cybersecurity.”
Network	A system of connected nodes. This concept, in other words, is defined similarly in the cybersecurity and organizational science domains. However, in the cybersecurity domain, the term is typically used to refer to computer (or more broadly information technology) networks consisting of computers connected to each other via data links, rather than to social networks consisting of employees connected to each other via communication media/modes.
Phishing attack	A type of social engineering attack where victims are directed to a fake website through a link, usually encountered in an email or other communication (e.g., text message) sent to them, or through websites they may encounter. The link, when clicked on (e.g., to access a website or download a file), may contain malware or may trigger further communications requesting personal information from the user (e.g., bank account or credit card numbers). A “spear-phishing” attack is a more sophisticated type of phishing attack that targets a specific individual (vs. a large set of recipients), generally by using publicly available information about that individual, thereby personalizing the email or other communication in an effort to enhance the likelihood of the individual clicking on the link.
Privacy	“The ability of individuals to understand and exercise control over how information about themselves may be used by others” (NICCS, 2018). This concept, in other words, is defined similarly in the cybersecurity and organizational science domains. For organizational science definitions, see Bhave et al. (2020).
Ransomware	Malicious software “that prevents or limits users from accessing their system,” for instance by encrypting the user’s files, until a payment (ransom) is paid (increasingly, via a cryptocurrency such as Bitcoin; Srinivas et al., 2019, p. 181).
Resilience	“The ability to adapt to changing conditions and prepare for, withstand, and rapidly recover from disruption” (NICCS, 2018). This concept, in other words, is defined similarly in the cybersecurity and organizational science domains. However, in the cybersecurity domain, the term is typically used to refer to the organization’s information technology networks (i.e., “resilient networks”) rather than to people (i.e., “resilient employees”).
Security	“[A] condition that results from the establishment and maintenance of protective measures that enable an enterprise to perform its mission or critical functions despite risks posed by threats to its use of information systems. Protective measures may involve a combination of deterrence, avoidance, prevention, detection, recovery, and correction that should form part of the enterprise’s risk management approach” (Cybersecurity glossary, n.d.).
Security Information and Event Management (SIEM)	“A formal process by which the security of an organization is monitored and evaluated on a constant basis. SIEM helps to automatically identify systems that are out of compliance with the security policy as well as to notify the IRT (Incident Response Team) of any security violating events” (Cybersecurity glossary, n.d.).
Security Operations Center (SOC)	An entity that serves to detect cyber incidents by triaging alerts generated by security information and event management software. The SOC subsequently forwards confirmed incidents to the CSIRT. Thus, the SOC is responsible for incident detection and the CSIRT for incident response (Barros, 2018). However, in many organizations, the functions of incident detection and incident response are handled by a single entity, which may variously be referred to as a SOC, a CSIRT, or something else (Barros, 2018; Ruefle, 2007). See also: Cybersecurity Incident Response Team (CSIRT).
Social engineering	“A non-technical technique that intrusion hackers commonly use. This approach relies on human interaction and often involves tricking people into breaking normal security procedures” (Cybersecurity glossary, n.d.). Thus, social engineering often involves the use of deception, manipulation, and persuasion on the part of attackers (Krombholz et al., 2015).
Spear-phishing attack	See “phishing attack.”
Spyware	A program that accesses a user’s personal information (e.g., email contact list, financial information stored on the computer, list of websites visited) and then transmits the information to an adversary for misuse (e.g., selling the smartphone contact list to a spammer; Srinivas et al., 2019).
Trojan horse	“A useful, or apparently useful, software program or command language that contains hidden code, that, when implored, executes some…undesirable or harmful function” (Srinivas et al., 2019, p.181).
Unauthorized access	An employee’s account information (e.g., his or her login credentials) is utilized by someone else to gain unauthorized access to a system (Srinivas et al., 2019).
Usable security	“[T]he usability of security tools (or *products*) and the *process* of designing secure systems for the real-world context (the *panorama*) in which they have to operate” (Sasse & Flechais, 2005, p. 13; emphasis in original). Usable security represents an alignment of research in the discipline of computer (or cyber) security with research on usability within the discipline of human-computer interaction.
Virus	“[A]n infectious program…[that] attaches itself to some other software (program) and reproduces itself when the software is executed by the user. Typically, [a] virus spreads through sharing of infected software or files among…computers and smartphones” (Srinivas et al., 2019, p. 181).
Worm	“A worm propagates itself from one system to another system, …actively [seeking] out more machines to infect [such that] each machine that is infected serves as an automated launching pad for attacks on other machines…. A worm can self-replicate and [spread] independently while a virus relies on some user action (such as running a program and opening of a file in a system)…to spread” (Srinivas et al., 2019, p.181). A worm“can destroy data and files” as well as “harm the host networks by consuming bandwidth and overloading the web servers” (Srinivas et al., 2019, p. 181).
Zero-Day Attack	A cyberattack that exploits a previously unknown security weakness in software (Frankenfield, 2020). Because the weakness was previously unknown to the software developer (let alone to organizations using the software), no patch for the weakness is yet available from the software developer—and the attack is therefore likely to damage organizational networks. The term originates from the number of days (viz., zero) the software developer was aware of the security weakness before it was exploited by attackers.

*Note.* For additional terms, see NICCS (2018) and Cybersecurity glossary (n.d.)

Given the “human factor” involved in so many cybersecurity events and given that so many cybersecurity events occur in organizational contexts where the human factor involves employees, we assert that organizational scientists should be at the forefront of studying employee behavior that leads to negative cybersecurity outcomes. There is a rapidly growing cybersecurity crisis in organizations (Dreibelbis, Martin, Coovert, & Dorsey, 2018), and this manuscript highlights how organizational scientists can best help with this challenge.^1^

Because the aim of this manuscript is to introduce a broad spectrum of organizational researchers to cybersecurity-relevant research, we have assumed very little prior knowledge of cybersecurity on the part of the reader. We moreover felt it important to cover a variety of topics rather than provide an in-depth treatment of a few topics. Accordingly, an important avenue for future research involves a series of narrower review papers targeted at individual topics within the broad domain surveyed here. Finally, although a variety of organizational science perspectives can (and should) fruitfully be brought to bear on cybersecurity, the authors of this manuscript possess expertise primarily in psychology and micro-organizational behavior—and it is therefore these perspectives that feature to a disproportionate extent in the manuscript. Accordingly, another important avenue for future research involves a companion overview paper from a macro-organizational science perspective.

We begin the focal part of this manuscript by defining organizational cybersecurity as well as key terms in organizational cybersecurity. Next, we illuminate the unique opportunities facing organizational scientists in their cybersecurity endeavors. Subsequent to this, we provide overall conceptual frameworks of the antecedents of employees’ cybersecurity behavior. In so doing, we focus not only on employees whose job formally involves deterring, detecting, and mitigating cyber threats to the organization, but also on the much larger number of “regular” employees, who, though not formally responsible for cybersecurity, may inadvertently or deliberately expose the organization to cyber threats. Because our goal is to motivate and facilitate organizational science research in the cybersecurity domain, we provide an expansive agenda for future organizational science research on cybersecurity—and we describe the benefits of such research not only to cybersecurity but also to organizational science itself. We end by providing a list of potential objections to such a research agenda, along with our responses to such objections.

## Definitions

According to ISO/IEC 27000 guidelines (2018), information security is defined as the “preservation of confidentiality, integrity and availability of information.” Confidentiality, integrity, and availability are commonly referred to in the security realm as the “CIA” triad. The CIA triad boils down to allowing authorized individuals access to complete, unaltered records (i.e., information) while simultaneously disallowing access to unauthorized individuals. In this definition of information security, there is no delineation between organizational information stored physically (e.g., notes made on paper) and electronically (e.g., personnel data stored on a server). There are debates in the literature regarding whether the terms information security and cybersecurity should be used interchangeably, as well as whether these terms should exclude information-based assets not stored or transmitted through information communication technology (e.g., von Solms & van Niekirk, 2013). However, an increasingly pervasive perspective holds that the cyber and physical worlds are converging, thereby necessitating the study of the security of information in cyber-physical systems (e.g., Rahman & Donahue, 2010). Accordingly, we define organizational cybersecurity broadly, as *the efforts organizations take to protect and defend their information assets, regardless of the form in which those assets exist, from threats internal and external to the organization* (see also Table 1).

Like any discipline, cybersecurity has spawned a large lexicon that serves as a barrier to entry by outsiders such as organizational science researchers. It is well beyond the scope of the current manuscript to provide an exhaustive summary of technical terms in cybersecurity. Nonetheless, Table 1 provides formal definitions for several cybersecurity terms that are particularly relevant to the human factor in cybersecurity and therefore to organizational scientists who wish to communicate with cybersecurity researchers and practitioners. We refer to many of these terms in the remainder of this manuscript, but we deliberately include additional terms in the table in order to illustrate both the nature of the cybersecurity discipline and the many potential entry points for organizational scientists.

## Unique opportunities for organizational science

The cybersecurity domain, as it pertains specifically to work organizations, presents organizational scientists with opportunities in terms of specific research directions and publication outlets. It also presents opportunities in terms of obtaining external funding for research. Finally, it presents opportunities in terms of studying two sub-populations of employees. We discuss these opportunities in turn.

## Opportunities in terms of research directions and publication outlets

In this section, we describe two broad avenues of research opportunities along with potential publication outlets associated with each avenue. In the remainder of this manuscript, we continue to use these two broad research avenues as an organizing framework for future research ideas.

The first broad avenue of organizational science research opportunity involves applying existing organizational science models, methods, and data-analytic techniques to the cybersecurity domain, and outlining the likely challenges of doing so. One way of thinking about this particular avenue of research opportunity is as a move from either “Bohr’s Quadrant” (pure basic research) or “Pasteur’s Quadrant” (use-inspired basic research) to “Edison’s Quadrant” (pure applied research; Stokes, 1997). Such a perspective would suggest a complete focus on application and a resulting dearth of novel basic research insights. However, we maintain that such an assertion would be inaccurate. Recent research in the area of “implementation science” (e.g., Al-Ubaydli, List, & Suskind, 2019), for instance, suggests that several factors related to the composition of the sample (e.g., selection bias, non-random attrition) and the nature of the research setting (e.g., situation selection, correct delivery and dosage) should be considered as potential moderator variables when scaling up research and applying it to a new domain—and therefore that, at its best, applied research can yield a deeper theoretical understanding of underlying phenomena and processes that, in turn, can feed back into basic research.

Research along these lines could be published in cybersecurity outlets (e.g., *IEEE Security & Privacy* and *Computers & Security*). With regard to outlets a bit closer to “home” (for organizational scientists), such research could be published in technology-focused journals with an organizational or social science bent. Of note, some of these journals have impact factors that are very strong by the standards of organizational science journals (e.g., both *Computers in Human Behavior* and *MIS Quarterly* have a 2019 journal impact factor above 5.00). Such research may, on occasion, also be publishable in top-tier organizational science journals if the research emphasizes theory elaboration (perhaps accounting for the sample and setting factors mentioned previously), theory integration (Bernard & Snipes, 1996), and/or competitive theory testing (Platt, 1964) as opposed to merely the “off-the-shelf” application of an existing organizational science model or measure to the cybersecurity domain. Overall, then, research categorized within the first broad avenue of opportunity offers the promise of a wider than usual array of publication outlets for organizational scientists.

The second broad avenue of research opportunity, in contrast, is aimed squarely at basic research in organizational science. If the first research avenue may be regarded as cross-fertilization from organizational science to cybersecurity, the second research avenue may be regarded as cross-fertilization in the opposite direction—thereby providing a novel “lens” with which to view traditional organizational science research topics. In the remainder of this manuscript, we propose several research topics that would involve cybersecurity-relevant contributions to basic research in the organizational sciences. For instance, we note that cybersecurity research can suggest important organizational phenomena that have been overlooked in existing organizational science research (e.g., collaboration triggering). Overall, due to its emphasis on facilitating basic research in the organizational sciences, research categorized within the second broad avenue of opportunity is likely to be publishable in top-tier organizational science journals.

## Opportunities in terms of obtaining external funding

Although many of the funding opportunities in cybersecurity remain focused on technical areas (e.g., networking and distributed ledger protocols, trusted hardware platforms, cryptography), an increasing number of opportunities involve sociotechnical-focused grants and contracts. Among those, the U.S. Department of Homeland Security (DHS), Defense Advanced Research Projects Agency (DARPA), Intelligence Advanced Research Projects Activity (IARPA), Army and Air Force Research Laboratories (ARL and AFRL, respectively), and Office of Naval Research (ONR) welcome multidisciplinary cybersecurity projects. Within the National Science Foundation (NSF), the Secure and Trustworthy Cyberspace (SaTC) program is a significant funding outlet, with other programs like Science of Organizations (SoO), Science and Technology Studies (STS), and Decision, Risk, and Management Sciences (DRMS) being applicable to social scientists in more discipline-specific cyber-relevant domains. International funding initiatives include the Security, Privacy, Identity and Trust Engagement NetworkPlus (SPRITE+) community and funding sources through the Australian Government, the National Cybersecurity R&D Programme through Singapore’s National Research Fund (NRF), the Academy of Finland, and Brazil’s National Research Network (RNP), to name just a few.

## Opportunities in terms of studying sub-populations of employees

The cybersecurity domain offers organizational scientists the opportunity to study two sub-populations of employees: end-users and cybersecurity-focused employees. In this section, we briefly describe each sub-population in turn. Subsequently, we continue to use these two sub-populations of employees as an organizing framework for the ideas we discuss in the remainder of the manuscript.

The first sub-population involves end-users in organizations, who are frequently referred to in the cybersecurity literature as organizational “insiders.” This sub-population involves all employees (full-time, part-time, temporary, and even external contractors) who interface with organizational information communication technology. For instance, leaks of National Security Agency (NSA) data by Edward Snowden (regarding global surveillance programs) and Reality Winner (regarding Russian interference in the 2016 U.S. elections) occurred while these individuals were employed by NSA subcontractors rather than directly by the NSA. We further distinguish between naïve end-users, who do not intend to harm the organization but who nonetheless have the potential to be victimized by cyberattacks, and malicious end-users, who intend to harm the organization and who have the potential to perpetrate cyberattacks.

The second sub-population involves cybersecurity-focused employees, whose formal job responsibilities include detecting and mitigating cybersecurity incidents as well as proactively “hardening” organizational computer networks against cybersecurity threats. Of course, such employees are themselves also end-users. Thus, the second sub-population is more accurately characterized as a specific subset of the first.

## Cybersecurity end-users

A primary goal for organizational scientists’ contribution to the cybersecurity domain is to try to minimize an end-user’s cyber misbehavior by understanding the organizational and individual difference factors that can predict such behavior as well as the design of interventions aimed at changing such behavior.

**Fig. 1:**
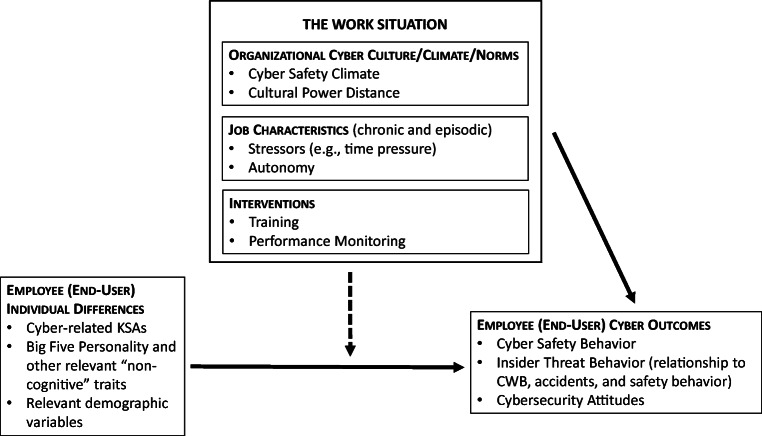
An Organizational Science Perspective on Behavioral Cybersecurity: End-User Model. *Note.* KSAs = Knowledge, Skills, and Abilities. CWB = Counterproductive Work Behavior

We organize the likely organizational science contributions to end-user (mis) behavior via Fig. 1. Before moving to a discussion of the components of Fig. 1, however, we issue three caveats. First, we view Fig. 1 merely as an organizing framework for future research rather than as a comprehensive psychological process model. Second, space constraints compel us to provide a selective, as opposed to comprehensive, discussion of the topics listed in Fig. 1. Third, although Fig. 1 emphasizes the contributions of organizational science topics to cybersecurity, our in-text discussion also covers knowledge transfer in the opposite direction (i.e., from cybersecurity to organizational science), which we believe is equally important. We summarize the future research agenda from our discussion of end-users in Table 2.

**Table 2:** Sample Future Research Needs at the Intersection of Organizational Science and Cybersecurity for *End-Users* of Cybersecurity

Broad Area for Future Research	Specific Suggestion for Future Research
Behavior or Performance	An urgent area for future research involves understanding the dimensionality of end-user cybersecurity performance. We must fully identify what we are attempting to predict before we can begin looking for predictors. One specific aspect of this broad problem is particularly noteworthy. The insider threat literature in cybersecurity varies in terms of whether accidental behavior is included under the rubric of insider threat behavior. In contrast, the organizational science literature is very consistent in making a distinction between accidental behavior and counterproductive work behavior. However, can counterproductive work behavior actually be distinguished from accidents either theoretically (i.e., in terms of different process models) or empirically (i.e., factor analytically and in terms of significantly different patterns of relationships with antecedents)? Where does counterproductive work behavior fit within broader classifications of unsafe acts (e.g., “slips,” “lapses,” “mistakes,” and “violations”; Reason, 1990)? Can we distinguish *behaviorally* (e.g., through latent behavioral profiles or “types” of employees)--as opposed to merely through antecedent-behavior relationships--between the clumsy, clueless employee who inadvertently causes a data breach and the “deviant” employee who intentionally leaks data to outsiders?
Attitudes	What is the dimensionality of end-user cybersecurity attitudes, and how can discrepant conceptualizations in this regard (e.g., Faklaris et al., 2019; Hadlington, 2017; Howard, 2018) be reconciled? Do the consequences (i.e., results or outcomes) of behavior influence the debate regarding causal direction in the attitude-behavior relationship—in other words, whether behavior predicts attitudes more strongly than attitudes predict behavior? Can “subjective norms” in the Theory of Planned Behavior (Ajzen, 1991) effectively be conceptualized as situational strength (Meyer et al., 2010) or organizational climate level and/or strength (Schneider et al., 2002), thereby integrating multiple research topics of interest to organizational science? Another important topic, which engenders a wealth of research questions (see the body of the paper for examples), involves the “cybersecurity calculus”: employees’ risk-benefit tradeoffs in the context of cybersecurity.
Habituation	Can a System 1 versus System 2 approach (Kahneman, 2011) effectively target routinized behavior as it pertains both to cybersecurity and to other forms of behavior important to organizational scientists?
Individual Differences	For naïve end-users, future research should examine the effect of a variety of demographic characteristics (e.g., age, gender, ethnicity) on vulnerability to cyberattacks separately from each other and from the effects of personality, risk-taking propensity, and cybersecurity knowledge, skills, and abilities—such that these latter constructs can be examined as potential mediators of any demographic differences in vulnerability to cyberattacks. For malicious end-users, future research should similarly tease out the differential effects of demographic characteristics (e.g., age) from correlated job characteristics (e.g., access and expertise) and cultural values (e.g., patriotism and civil disobedience). With regard to employee age specifically, future research should examine the full range of age observed in the workplace (but also not an overly wide age range that would include children and retirees) and should avoid underpowered studies. Preliminary research suggests, somewhat surprisingly, that the personality trait of conscientiousness may not stand out as a consistent predictor of naïve end-user susceptibility to cyberattacks. Future process-oriented primary studies using stronger research designs should continue to explore this question. Does cybersecurity training for naïve end-users—aimed at improving knowledge, skills, and abilities—actually increase risk calibration (the desired outcome) or merely risk aversion (an undesired outcome, with likely negative consequences for task performance)? Based on findings regarding the role of technical expertise on malicious end-user cyber misbehavior, future organizational science research should examine the role of technical expertise in various forms of counterproductive work behavior. Perhaps existing organizational science taxonomies of counterproductive work behavior (e.g., Robinson & Bennett, 1995) should be augmented by an additional dimension distinguishing between behavior that does versus does not require technical expertise to execute (or execute *successfully*). Do findings regarding the impact of naïve end-user individual differences on cyber behavior generalize across research designs? Organizational science researchers can adopt research designs from cybersecurity research on malicious end-user behavior. For instance, counterproductive work behavior can be studied through an analysis of a set of historical case studies (see Yin, 2017) and through the use of “honeypots.” Because existing research on naïve end-users has focused primarily on individual differences in the context of phishing attacks, future research should study whether these findings generalize to other types of cyberattacks (e.g., ransomware, Trojan horses; see Table 1 for definitions of these terms).
The Work Situation	With regard to organizational policies and climates, research should continue to explore connections between cybersecurity and occupational safety. Cybersecurity research can fruitfully continue to modify measures from the occupational safety literature. Conversely, occupational safety research can benefit from modifying formal models and misuse case diagrams from the security literature. The cybersecurity domain, with its tension between ancillary versus core demands as well as its identification of the importance of “non-events” (e.g., cyberattacks that did not occur due to the employee’s actions) may suggest the need for extensions to Job Characteristics Theory (Hackman & Oldham, 1976) and other job design theories. Organizational training in areas such as diversity training and sexual harassment prevention training could benefit from the common distinction in cybersecurity between compliance with regulations (i.e., a checklist culture) and actual risk or harm mitigation. Unlike in many organizational settings, where behavior is difficult to measure and therefore intervention effectiveness is assessed primarily through knowledge tests and attitude measures, many cyber behaviors (e.g., password changes) are documented automatically through the organization’s information technology system. Thus, cybersecurity training could allow more robust tests of training intervention approaches across time, thereby generating new insights into decay of training, reactance, and differential effectiveness (including aptitude-treatment interactions).

### Behavior

Willison and Warkentin (2013) categorize human threats to cybersecurity along a continuum from “passive, non-volitional noncompliance” (e.g., forgetful oversights) to “volitional (but not malicious) noncompliance” (e.g., failing to log off when leaving computer) and finally to “intentional, malicious (harmful) computer abuse” (e.g., data theft). In addition, as discussed in more detail in the context of the antecedents to behavior (covered in subsequent sub-sections), end-user cyber (mis) behavior exhibits important connections to organizational science research topics such as counterproductive work behavior, unintentional negligent (i.e., accidental) behavior, and physical safety behavior (Chan, Woon, & Kankanhalli, 2005; Dalal & Gorab, 2016). In other words, much like employee performance per se, end-user cybersecurity performance has a “criterion problem” (Austin & Villanova, 1992): performance (i.e., behavior relevant to organizational goals) is much less studied than its presumed antecedents. An urgent area for future research therefore involves understanding the dimensionality of end-user cybersecurity performance. We must fully identify what we are attempting to predict before we can begin looking for predictors. With that stipulation in mind, we begin our discussion of potential antecedents to end-user cybersecurity performance.

### Attitudes

In the organizational sciences, job attitudes are viewed both as a means to an end (i.e., a mechanism for increasing job performance) and, to a lesser extent, as an end (or outcome) in and of themselves. Job attitudes research is among the most popular topics in organizational science (Judge & Kammeyer-Mueller, 2012). Furthermore, one of the most empirically supported theories is Ajzen’s (1991) Theory of Planned Behavior, which states that attitudes toward behavior, along with perceived behavioral control and social norms, predict behavioral intentions, which in turn predict behavior. In an organizational context, attitudes such as job satisfaction have been found to be valid predictors of overall job performance (Judge, Thoresen, Bono, & Patton, 2001), organizational citizenship behavior (Dalal, 2005), and counterproductive work behavior (Dalal, 2005). Thus, employees’ attitudes toward cybersecurity are likely to inform organizations about subsequent cyber-related behavior.

Although job attitudes have been a prevalent research topic for organizational scientists, there is much less research on attitudes toward cybersecurity policies and procedures. Some existing work in the information systems literature has focused on information security perceptions and the adoption of cybersecurity policies by organizations or their employees using the Technology Acceptance Model (e.g., Johnson, 2005; Jones, McCarthy, Halawi, & Mujtaba, 2010). This model posits that perceived ease of use and perceived usefulness are key in the adoption of technology (Davis, 1989). Though attitudes and perceptions are not identical constructs, they are closely related (Pickens, 2005). Borrowing from the information scientists, organizational scientists should consider perceptions of usefulness and ease of use when measuring attitudes toward cybersecurity policies and procedures. Moreover, organizational scientists should take note of additional (beyond the Technology Acceptance Model) perspectives on attitudes toward cybersecurity (see, e.g., Herath & Rao, 2009; Ifinedo, 2014).

There is also much work to be done when measuring cybersecurity attitudes as a separate set of constructs. For instance, three recent measures of end-user cybersecurity attitudes (Faklaris, Dabbish, & Hong, 2019; Hadlington, 2017; Howard, 2018) vary appreciably in the number of obtained facets and in terms of whether cybersecurity should be conflated with cybercrime. These disagreements regarding factor structure and outcomes are similar to disagreements involving other job attitudes, such as job satisfaction (e.g., Dalal & Credé, 2013).

We propose the cybersecurity domain as fertile ground for applying and extending organizational science theory regarding job attitudes. Frequently, in the end-user cybersecurity domain, an individual’s intention and behavior lead to outcomes that are unexpected and of a much larger magnitude than imagined. If, for example, an employee is the victim of a spear-phishing attack (see Table 1 for a definition) that leads to serious consequences for the organization, does the individual experience cognitive dissonance, such that the employee’s behavior leads to attitude change? Researchers could use longitudinal designs to compare and contrast the attitude-to-behavior link predicted by the Theory of Planned Behavior with the behavior-to-attitude link predicted by Cognitive Dissonance Theory (Festinger & Carlsmith, 1959) and Self-Perception Theory (Bem, 1967). Such research would be helpful to organizational science, which has generally assumed an attitude-to-behavior sequence even though this assumed causal direction has periodically come under withering criticism from both within and beyond the organizational sciences (e.g., Judge et al., 2001).

The study of cybersecurity attitudes would also benefit from the incorporation of additional belief-based constructs. For example, the other components of the Theory of Planned Behavior (i.e., perceived behavioral control and subjective norms) could be adapted to the cybersecurity domain (for initial attempts, see Aurigemma & Mattson, 2017; Cox, 2012; additionally, for information control from an invasion of privacy perspective, see Stone-Romero & Stone, 2007). Doing so would lead to research questions involving alternative conceptualizations of these constructs (e.g., conceptualizing subjective norms as situational strength or as organizational climate strength: Meyer, Dalal, & Hermida, 2010; Schneider, Salvaggio, & Subirats, 2002) that would inform organizational science research as well.

As another example, employees’ risk-benefit tradeoffs, previously studied occasionally by organizational scientists and more frequently by others (economists, legal scholars, etc.) in the context of privacy (i.e., the “privacy calculus”; Acquisti, Brandimarte, & Loewenstein, 2015; Bhave, Teo, & Dalal, 2020; Klopfer & Rubenstein, 1977), could be imported into the cybersecurity domain (i.e., the “cybersecurity calculus”). Doing so would generate a wealth of important research questions regarding the interplay between perceived risks and perceived benefits—a topic that is not well understood in any domain, let alone cybersecurity. For instance, does the impact of perceived risks on cybersecurity outcomes depend on the impact of perceived benefits, and vice versa (an interactive relationship)? If levels of perceived risks and perceived benefits are in alignment for an employee, does end-user cybersecurity behavior differ during occasions when the levels of both risks and benefits are high versus occasions when they are both low? Research questions such as these do not merely involve an application of organizational science to cybersecurity; rather, they are also of fundamental interest (and novelty) to organizational science itself. In particular, the research questions proposed in this paragraph are an example of *within*-person consistency or fit, which is greatly understudied in organizational science research in contrast to person-person (i.e., *between*-person) fit and, even more commonly, person-*environment* fit.

### Habituation

Beyond attitudes, another area relevant to employees’ cyber behavior is habituation. Defined as “decreased response to repeated stimulation” (Groves & Thompson, 1970, p. 419) or routinized behavior (Vance, Siponen, & Pahnila, 2012), habituation has been shown to be detrimental to organizational security. The impact of habituation becomes ever more prominent as organizations steadily increase the number of informational cues employees receive daily via new technologies designed to increase efficiency and effectiveness (Deloitte, 2018). Despite their benefits, productivity systems such as email can: (1) cause fatigue, stress, and overload, (2) lead to habituation and lack of attention, and thus (3) become an entry point for employees’ detrimental cyber behaviors (Pfleeger & Caputo, 2012; Vishwanath, 2016; Vishwanath, Herath, Chen, Wang, & Rao, 2011; Wainer, Dabbish, & Kraut, 2011).

Interestingly, cybersecurity efforts, demands, and techniques can themselves lead to habituation and harmful employee reactions. For example, recent neuroscience efforts in cybersecurity have determined that habituation sets in after only a few exposures to information security warnings (Anderson, Jenkins, Vance, Kirwan, & Eargle, 2016), and that interrupting an employee with security warnings during other cognitive tasks often leads to the employee disregarding the warnings (Jenkins, Anderson, Vance, Kirwan, & Eargle, 2016). Likewise, organizational cyber demands can lead to overload (D’Arcy, Herath, & Shoss, 2014) and overly frequent organizational emails regarding cybersecurity issues can lead to habitual inattention to and/or wanton deletion of those emails (Posey, Roberts, Lowry, & Hightower, 2014).

One exciting paradigm that can help shed light in this area is the psychology and behavioral economics research on System 1 versus System 2 thinking (Kahneman, 2011). Though many cyber behavioral theories explicitly rely on foundations like rational choice and cost-benefit differentials, this perspective is likely incomplete and potentially even actively misleading, given that much of what we are interested in regarding employees’ cyber behaviors is affected by System 1 operations (i.e., automatic, quick operations with little effort) in addition to and in some cases instead of System 2 operations (i.e., slow, effortful mental actions; Dennis & Minas, 2018). Thus, many employees’ cyber-relevant behaviors might not be intentional but rather an outcome of a routinized or heuristical way of operating in the workplace.

This research area is in its infancy. Although the existing literature shows that both polymorphic warnings (i.e., pop-up warning messages intentionally designed to change in appearance across iterations) and the introduction of an intentional recovery period wherein individuals are not further exposed to the warnings for that day (Vance, Jenkins, Anderson, Bjornn, & Kirwan, 2018) can decrease the rate of habituation, much more research is needed. For example, which forms of employee habitual or routinized behavior are likely to result in the greatest exposure to cyber threats? Further, if we expect employees to increase their System 2 thinking for their daily decisions even among short, repetitive tasks, organizations must be willing to decrease the productivity expected from their employees. This tradeoff is currently being explored through computational social science approaches (Posey & Canham, 2018). Importantly, such research efforts (e.g., habituation to events, security-productivity tradeoffs), though conducted within the domain of cybersecurity, are both novel to and contribute to our understanding of fundamental questions in organizational science.

### Individual differences predictors

In this section, we discuss individual differences in end-users that are likely to predict end-user outcomes. We distinguish between naïve and malicious end-users.

#### Naïve end-users

Most existing research examining the impact of individual differences on susceptibility to cyberattacks has focused on one specific type of cyberattack: namely, the phishing attack (see Table 1 for a definition). Phishing attacks are very common (e.g., as early as April 2020, Google’s Threat Analysis Group detected and blocked “18 million malware and phishing Gmail messages per day related to COVID-19,” with examples that included “messages that try to mimic employer communications to employees working from home”; Huntley, 2020), but they are by no means the only type of cyberattack. Future research should therefore adapt existing organizational science models and measures to the examination of the impact of individual differences on other types of cyberattacks (e.g., ransomware, Trojan horses; see Table 1 for definitions). Given the body of existing research, however, in this section we focus our attention on individual differences in vulnerability to phishing attacks in particular. Specifically, we discuss demographic, non-cognitive (i.e., personality), and cognitive (i.e., knowledge, skills, and abilities) individual difference antecedents to end-user vulnerability to phishing attacks.

In terms of employee *age*, research often (e.g., Darwish, El Zarka, & Aloul, 2012; Diaz, Sherman, & Joshi, 2020; Kumaraguru, Sheng, Acquisti, Cranor, & Hong, 2010; Sheng, Holbrook, Kumaraguru, Cranor, & Downs, 2010) appears to suggest that age is negatively correlated with susceptibility to phishing attacks, with the effect being driven by high susceptibility to phishing attacks in the late teen years and early twenties. The direction of this relationship may at first seem surprising, given lay beliefs that older employees, who have had to learn to interact with technology in adulthood (“digital immigrants”), may not possess the same technological expertise as younger employees, who have grown up with technology (“digital natives”; Prensky, 2013). One possibility is that the result is due to young adults engaging in more risky behavior than older adults, though meta-analytic results suggest that the relationship between age and risk-taking is nuanced (i.e., task- and frame-dependent; Defoe, Dubas, Figner, & Van Aken, 2015; Mata, Josef, Samanez-Larkin, & Hertwig, 2011).

In addition, it is important to understand that, oftentimes, studies examining the age-vulnerability relationship have surveyed populations: (1) possessing a relatively narrow range of technological expertise (such as university students, craigslist.com respondents, and Amazon.com’s Mechanical Turk workers), and/or (2) containing few older or even middle-aged individuals (e.g., only 7% of Kumaraguru et al.’s, 2010, respondents were older than 34 years of age; all of Diaz et al.’s, 2020, respondents were undergraduate students). Thus, the studies that assessed age-victimization relationships often: (1) indirectly controlled for technological expertise, and (2) used severely range-restricted age scores. In contrast, Oliveira et al. (2017) did study a sub-population of older individuals (mean age = 72 years) and found that, in comparison to another sub-population of younger individuals (mean age = 22 years), the older individuals were more susceptible to spear-phishing attacks. However, the sub-population of older individuals in the Oliveira et al. study would, for the most part, no longer be in the workforce. Overall, then, the impact of age on phishing susceptibility in employee samples remains unclear.

In terms of employee *gender*, research often but not always suggests that women are more susceptible than men to being phished (e.g., Darwish et al., 2012; Gratian, Bandi, Cukier, Dykstra, & Ginther, 2018; Halevi, Lewis, & Memon, 2013; Sheng et al., 2010; but see van de Weijer & Leukfeldt, 2017, who found that men are more susceptible), though the reasons for this gender difference are not entirely clear. Finally, there is a paucity of research on possible *race/ethnicity*-related differences in phishing susceptibility, perhaps due to the paucity of theoretical reasons to expect such differences.

Overall, then, future research on vulnerability to phishing attacks should simultaneously examine the effect of a variety of demographic characteristics (e.g., age, gender, ethnicity) along with technological expertise (or cybersecurity knowledge, skills, and abilities more specifically), risk-taking propensity, and personality—such that these latter constructs can be examined as potential mediators of any demographic differences in vulnerability. With regard to employee age specifically, future research should examine the full range of age observed in the workplace (but also not an overly wide age range that would include children and retirees) and should avoid underpowered studies (cf. Kumaraguru et al., 2010). Organizational science researchers, with their understanding of direct and indirect range restriction as well as their somewhat greater attention to sampling and statistical power, could contribute greatly to such research.

In terms of employee *personality*, research reveals mixed findings across studies (Darwish et al., 2012; Lawson, Crowson, & Mayhorn, 2018). Given the organizational science research on personality, with conscientiousness being the “go to” personality construct for most forms of behavior of value to the organization (e.g., Barrick & Mount, 1991; Christian, Bradley, Wallace, & Burke, 2009; Dalal, 2005), it seems surprising that conscientiousness does not stand out as a consistent predictor of phishing susceptibility. Perhaps research using stronger research designs would reveal an effect of conscientiousness. If not, then perhaps more nuanced organizational science theories are needed to predict the specific forms of employee behavior that are influenced by conscientiousness—and the process through which this effort occurs.

In terms of cybersecurity *knowledge, skills, and abilities* (KSAs), the National Initiative for Cybersecurity Education’s Cybersecurity Workforce Framework (Newhouse, Keith, Scribner, & Witte, 2017) lists 630 knowledge items (e.g., “Knowledge of the importance of ingress filtering to protect against automated threats that rely on spoofed network addresses,” p. 76), 374 skills (e.g., “Skill to respond and take local actions in response to threat sharing alerts from service providers,” p. 87), and 176 abilities (e.g., “Ability to find and navigate the dark web using the TOR network to locate markets and forums,” p. 94). As illustrated by the examples provided, many of these KSAs are quite technical and therefore uncharacteristic of laypersons such as the vast majority of naïve (vs. malevolent) end-users. Perhaps unsurprisingly, therefore, the effect of cybersecurity KSAs on naïve end-users has typically been studied through cybersecurity end-user training (see, e.g., Al-Daeef, Basir, & Saudi, 2017), which is generally relatively simple and rule-based (e.g., teaching trainees to look for encrypted connections to websites via cues such as HTTPS in the address bar and a lock icon in the browser; Jensen, Dinger, Wright, & Thatcher, 2017) and which is often grounded in Signal Detection Theory (Martin, Dubé, & Coovert, 2018).^2^

An intriguing question, however, is whether training actually increases accuracy of distinguishing phishing emails from legitimate ones—in other words, risk calibration. It is possible that training instead simply increases risk aversion, such that trained participants become more suspicious of *all* emails: that is, not just phishing emails but also legitimate emails (Al-Daeef et al., 2017; Sheng et al., 2010). Increased risk aversion across the board is likely to result in a large number of false positives (i.e., classifying legitimate emails as phishing threats), thereby impeding job performance. Future research could explore this question in more detail, with an emphasis on identifying features of training programs that increase accuracy rather than risk aversion. The implications of such research are important to other forms of training relevant to organizational science—for example, safety training. To our knowledge, existing safety training research in the organizational sciences has not emphasized the importance of fostering risk calibration rather than blanket risk aversion.

It is also noteworthy that the studies cited in this section generally use variations on two types of research designs. One such research design exhibits some similarity to a work sample in-basket (or inbox) test. Participants are aware that they are participating in a research study and are presented with a set of emails, of which some are legitimate whereas others are phishing emails. Participants then categorize each email as legitimate versus phish either explicitly or else implicitly—in the latter case, through their responses to each email in a role-play situation (e.g., reply to email vs. delete email vs. click on potentially malicious link in email). In contrast, the second research design is in situ. Participants are unaware that they are participating in a research study and are sent phishing emails generated by the researchers. If participants click on a link in a phishing email, they are directed to a website maintained by the researchers (as opposed to a real phishing website) and are alerted to the fact that they have clicked on a phishing email generated as part of a research study.

Given the differences between the two research designs, the paucity of research on the impact of research design features on findings is notable. More broadly, there appears to be very little existing research on situational moderators of the relationship between individual differences and phishing susceptibility, thereby presenting an important opportunity for future research. For instance, the aforementioned research designs may differ in terms of some of the potential task-related moderators in Fig. 1 (e.g., time pressure, autonomy, and, in cases where emails appear to originate from one’s supervisor, power distance). Future research could evaluate the two research designs explicitly in terms of user perceptions of multiple task-related features, such as those in Fig. 1.

#### Malicious end-users

Little research attention has been paid to the individual characteristics of malicious insiders, perhaps because of the low base-rate of malicious insider attacks (or the lack of reporting of such attacks by organizations not wanting the negative exposure; King, Henshel, Flora, Cains, Hoffman, & Sample, 2018). However, limited research on age or generation shows that “millennials”—members of a younger generation—appear to be no more likely to become insider threats than members of older generations; in fact, some data show that they are less likely to do so than members of “Generation X” (Fisher, 2015). If indeed older generations of employees do engage in more malicious insider behaviors than younger generations, that would be contrary to the general organizational science finding that age is related negatively to counterproductive work behavior (for a meta-analysis, see Ng & Feldman, 2008) and may be attributable less to the aging process itself and more to the fact that access and expertise increase as employees advance in their careers. Cultural values of patriotism and civil disobedience, which may be related to age, have also been suggested as factors that influence insiders’ willingness to engage in cyber misbehavior (Hathaway & Klimburg, 2012, cited by King et al., 2018). For instance, Chelsea Manning invoked such values to justify the leaking of classified information to Wikileaks (Fortin, 2019). Future research should carefully tease apart demographic and cultural/political/work values as potential antecedents to malicious insider behavior so as to determine whether they withstand careful evaluation with controls in place.

Because malicious insider behavior is a form of counterproductive or deviant work behavior, it is likely to be influenced by a similar set of antecedents. In this regard, it is particularly interesting to consider the case of KSA antecedents to malicious insider behavior. As noted previously, many cybersecurity KSAs are highly technical and beyond the reach of naïve end-users. With regard to malicious end-users, in contrast, Venkatraman, Cheung, Lee, Davis, and Venkatesh’s (2018) multidimensional scaling (MDS) analysis revealed that cyber-deviant behaviors can be classified as: (1) minor versus serious, (2) those that target individuals versus organizations, and, importantly, (3) those that require low versus high technical expertise. The former two dimensions validate Robinson and Bennett’s (1995, 1997) original workplace deviance taxonomy that has been used extensively in organizational science research. Venkatraman et al.’s (2018) third dimension (low vs. high technical expertise), however, is not captured in Robinson and Bennett’s (1995) original taxonomy or in other organizational science taxonomies of counterproductive or deviant work behavior. Yet, in the cybersecurity literature on insider threat, Willison and Warkentin (2013) suggest—in a similar vein to Venkatraman et al. (2018)—that “[malicious] insiders are employees who have: (1) access privileges and (2) intimate knowledge of internal organizational processes that may allow them to exploit weaknesses” (p. 2). The knowledge into internal organizational processes that Willison and Warkentin (2013) describe is akin to technical expertise.

This emphasis on technical expertise in the cybersecurity literature may reveal an important gap in the organizational science literature. Specifically, technical expertise is necessary not only for some forms of cyber-deviance but also for some forms of counterproductive or deviant behavior in other domains: for instance, financial fraud. In fact, technical expertise may be required to execute sophisticated examples of even common forms of counterproductive work behavior (Spector, Fox, Penney, Bruursema, Goh, & Kessler, 2006) such as work withdrawal, production deviance, and interpersonal abuse. The nil meta-analytic relationship between general mental ability and counterproductive work behavior (corrected correlation = −0.02; Gonzalez-Mulé, Mount, & Oh, 2014) may therefore reflect: (1) the omission, from common measures of counterproductive work behavior, of behavior requiring technical expertise, and/or (2) a potentially misguided focus on general mental ability as opposed to technical expertise as a predictor of counterproductive work behavior. Alternatively, instead of influencing the frequency with which counterproductive work behavior is enacted per se, technical expertise may influence the frequency with which such behavior is enacted *successfully* (i.e., without being identified or even detected). Overall, then, this may be an area where cybersecurity insights spill over into broader organizational science research, revealing a potentially mistaken assumption in the latter that technical expertise does not matter in the (successful) execution of counterproductive work behavior.

Finally, as with naïve end-users, it is important to consider research designs used to study malicious end-users. In this regard, one concern is that malicious end-user behavior may be an even lower base-rate phenomenon than, say, naïve end-user behavior associated with falling prey to phishing attacks. Thus, uncovering malicious insider behavior can be a time-consuming endeavor—and one that may require novel (to organizational science) research designs. For example, Maybury et al. (2005) summarized a collaborative, six-month Advanced Research and Development Activity Northeast Regional Research Center challenge workshop that analyzed past and projected cases of sophisticated malicious insiders in the United States Intelligence Community in order to improve future detection and deterrence. They began with a qualitative analysis of historical cases, summarizing causal factors such as position, motive, computer skill, polygraph experience, prior cybersecurity violations, counterintelligence activities, and physical and cyber access. They then simulated malicious insiders with these traits to determine whether the security system would flag their behavior. Another design they described involved honeypots (see Table 1 for a definition) that enticed malicious insiders to entrap themselves by accessing a seemingly tempting (but actually fictitious) target.

The former example in the previous paragraph suggests that, to effectively study very low base-rate forms of counterproductive work behavior, organizational science researchers could make more use of case studies (e.g., from legal judgments and organizational disciplinary files). A single case study may be of limited use due to its idiosyncrasies, but a set of case studies can reveal regularities—and we, like Dalal and Gorab (2016), advocate that organizational science researchers contrast theoretically expected patterns of antecedents with empirical patterns observed in the set of case studies (see Yin, 2017, and, for an example from the insider threat literature, see Moore, Hanley, & Mundie, 2011). The latter example in the previous paragraph, on the other hand, suggests that organizational scientists could broaden their horizons when thinking about how to detect and deter counterproductive work behavior. Specifically, organizational scientists could work with cybersecurity practitioners to develop honeypots and make them enticing to those insiders considered to be at high risk for engaging in malicious behavior. In fact, given that the original use of the term “honey pot” or “honey trap” was not cyber-related at all (rather, it reflected romantic or sexual entrapment; see, e.g., Knightley, 2010), it may even be possible for organizational scientists to develop honeypots for other, non-cyber forms of counterproductive work behavior (e.g., theft of money or supplies).^3^

Beyond the case study design, other qualitative methods can also be utilized by scientists examining both malicious and non-malicious cyber behavior. Posey et al. (2014) conducted semi-structured interviews with both information security professionals and other professionals in examining the differences in mindset regarding organizational information security efforts between the two subgroups of employees. Posey et al. found that, although there was some consistency between the two groups, there were major differences in mindset regarding positive, protective employee behaviors. The interviews allowed for a more nuanced discovery of differences in attitudes beyond what a quantitative study might allow. Crossler, Johnston, Lowry, Hu, Warkentin, and Baskerville (2013) further recommend the use of grounded theory as a potentially effective method to better understand the motivations and behaviors of end-users.

Furthermore, we contend that mixed-methods research approaches could be beneficial in helping organizational scientists unravel the inherent complexity in how individual differences in both malicious and non-malicious end-user behavior interact with the work situation in predicting outcomes. Flores, Antonsen, and Ekstedt (2014) used a mixed-methods approach in discovering cultural differences between the U.S. and Sweden on information security governance factors’ effect on subsequent information security knowledge sharing. The authors first conducted interviews and analyzed the qualitative data to conceptualize the constructs to be measured and developed hypotheses to test in the resulting quantitative study. This method can be referred to as a sequential exploratory mixed-methods design (i.e., qualitative ➔ analysis ➔ quantitative ➔ interpretation of results). Other mixed-method designs include a sequential explanatory design (i.e., collecting quantitative data that informs a subsequent qualitative study), and concurrent data collection designs (e.g., triangulation). Any of the mixed-method designs might prove valuable to organizational scientists and cybersecurity researchers, because they can be utilized where complex situations require a more in-depth approach.

### The work situation

The work situation is multifaced and can be viewed through multiple lenses—for instance, those pertaining to organizational policies and climates, job characteristics and job design, and interventions. We discuss each topic in turn.

#### Organizational policies and climates: Lessons from, and contributions to, the organizational safety literature

An organizational science domain that has much in common with cybersecurity is occupational safety, with both domains emphasizing the minimization of loss through the reduction of accidents and errors. Furthermore, adherence to both safety and cybersecurity policies frequently creates an inconvenience for the employee, and some aspects of task performance can suffer as a result (Chan et al., 2005). To illustrate, employees not wanting to wear safety equipment because it is uncomfortable or hot or takes too long to put on could be likened to employees not wanting to remember a unique password for each software application they use. Cybersecurity policies can act as organizational constraints on the individual and can therefore be perceived as stressors. Thus, many occupational safety and occupational health psychology models aimed at predicting safety behavior could be applied to cybersecurity research. For example, the end-user framework proposed in this manuscript (Fig. 1) is in many ways akin to Neal and Griffin’s (2004) safety model that has obtained strong empirical support. Authors of information security climate instruments have noted the similarities between workplace safety and information security, and have in fact adapted safety scales into information security measures (Chan et al., 2005; Kessler, Pindek, Kleinman, Andel, & Spector, 2019). Conversely, occupational safety research can benefit from prior work conducted in the security realm. For instance, Piètre-Cambacédès and Bouissou (2013) highlight the adaptation of formal security models to analyze high-impact safety issues and the possibility of employing misuse case diagrams to elucidate safety concerns and requirements (e.g., Sindre, 2007).

#### Job characteristics and job design

Given the aforementioned concerns related to overload, demand, and habituation and their influence on employees’ cyber behaviors, researchers may need to examine to what degree the components of Job Characteristics Theory (Hackman & Oldham, 1976) apply in the modern workplace where cybersecurity demands and constraints are at times at odds with one’s traditional job roles. Specifically, future research could more deeply investigate how employees’ “ancillary” cybersecurity-relevant demands interact with the “core” job-task demands with which they can interfere (Posey et al., 2014; Post & Kagan, 2007).

As a reminder, Job Characteristics Theory’s job dimensions include skill variety, task identity, task significance, autonomy, and feedback. Regarding skill variety, modern employees need to continuously acquire new and varied skill sets to perform not only their core job tasks (e.g., increased reliance on data analytics) but also, simultaneously, fairly extensive and complex cyber-relevant demands (Burns, Posey, & Roberts, 2019; Posey, Roberts, Lowry, Bennett, & Courtney, 2013). The task identity and task significance components of the Job Characteristics Theory appear to align well with employees being able to ascertain whether their cybersecurity behavior really makes a significant impact. Unfortunately, these employee beliefs are not always easily formed in situ for at least two reasons. First, cybersecurity is a domain in which it is nearly impossible to determine how many cyberattacks did *not* occur during a given time period as a result of employees’ appropriate actions (i.e., “dynamic non-events”; Weick, 1987). Second, consider employees’ cyber coping appraisals. These include self-perceptions regarding their ability to perform recommended actions (i.e., self-efficacy) and their beliefs that the recommended actions are effective at reducing organizational risks due to cyber threats (i.e., response efficacy). Cyber coping appraisals demonstrate moderately strong effects on cyber behavior such as compliance with corporate information security policies; yet, organizational security education, training, and awareness efforts are not always designed to enhance or calibrate such appraisals (Cram, D’Arcy, & Proudfoot, 2019).

The two characteristics described in the Job Characteristics Theory that help build employee responsibility—namely, autonomy and feedback—also appear to be at odds with current organizational cyber operations. Due to cybersecurity concerns (among others), organizations are hesitant to allow employees the freedom to do their job tasks in the way they deem most appropriate. This leads to issues of shadow information technology and policy workarounds as employees attempt to get core tasks completed despite cyber controls (Blythe, Koppel, & Smith, 2013; Posey & Canham, 2018; Silic & Back, 2014). Also, pointing once again to the dynamic non-event nature of cybersecurity, managers have difficulty providing meaningful, positive feedback regarding relatively invisible employee protective behaviors (e.g., double-checking the addresses in an email prior to pressing the “send” button) that result in the non-occurrence of adverse events. Rather, it is when an adverse cyber event does occur due to employee actions (e.g., clicking on links in phishing emails) that such actions are more easily included in performance feedback to the employee. These examples suggest potential boundary conditions to the applicability of Job Characteristics Theory, and job design theories more generally, in the modern workplace.

#### Interventions for end-users

The main vehicle organizations utilize to influence employees’ cybersecurity behavior is security education, training, and awareness programs. The goals of such programs include making employees aware of existing threats to the organization, training employees to perform their cybersecurity roles, and discussing the content of organizational information security policies (Burns, Roberts, Posey, Bennett, & Courtney, 2018; D’Arcy, Hovav, & Galletta, 2009; Straub & Welke, 1998). At least in the U.S., organizations have largely relied on checklists derived from various governmental and industry specifications (e.g., the Federal Information Security Management Act) to inform their decisions regarding employee training, and both the content of these interventions and the frequency with which they are deployed are far from standardized (Cobb, 2018; Dehoyos, 2019; Madon, 2018). A sole focus on complying with government and industry mandates creates a checklist compliance culture (Moniz, 2018); compliance is not synonymous with risk or harm mitigation (Burns et al., 2018).

This area therefore provides a unique opportunity for research on the effectiveness of training and other behavioral interventions. For example, one question for future research involves how to effectively transition an organization steeped in a “checklist compliance” culture to one actively and iteratively attempting to improve protection of important organizational assets. Moreover, because cybersecurity is often viewed as an ancillary rather than a core job function, another question involves the optimal content, approach, and frequency of organizational security education, training, and awareness interventions. Research on questions such as these has the potential to benefit not just cybersecurity training but also other training efforts studied by organizational scientists that may operate within a suboptimal regulatory compliance (vs. actual risk or harm mitigation) culture: for instance, diversity training and sexual harassment prevention training.

In addition, intervention research in the organizational sciences is often limited by the ability to accurately and efficiently measure the behavior the interventions are designed to influence. For this reason, much of our knowledge of the effectiveness of interventions is based not on behavior but instead on knowledge tests and attitude measures. However, some of the behaviors included in the training objectives for cybersecurity are relatively easy to measure because they include concrete behaviors, such as changing a password, that are documented automatically through the organization’s information technology system (e.g., do employees volitionally change their passwords more frequently than required by the organization—and, when they do so, how different are their old and new passwords?). The advantage of ready access to automatically documented behavioral outcomes could allow more robust tests of training intervention approaches, thereby generating new insights into decay of training, reactance, and differential effectiveness (including aptitude-treatment interactions). If organizational scientists become more knowledgeable about cybersecurity training and the outcomes that can be captured automatically, cybersecurity training could serve as a fertile test bed for organizational science training research per se.

## Cybersecurity-focused employees

**Fig. 2:**
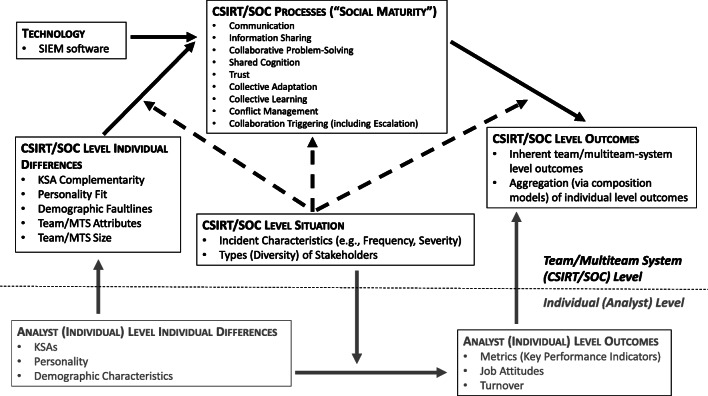
An Organizational Science Perspective on Behavioral Cybersecurity: Cybersecurity-Focused Employee Model. *Note.* KSAs = Knowledge, Skills, and Abilities. MTS = Multiteam System. CSIRT = Cybersecurity Incident Response Team. SOC = Security Operations Center. SIEM = Security Information and Event Management

Figure 2 depicts an organizing framework for the organizational science study of cybersecurity-focused employees. These employees frequently work in Cybersecurity Incident Response Teams (CSIRTs) or Security Operations Centers (SOCs), entities defined in Table 1. Because there is little agreement on this issue in the cybersecurity literature itself, we hereafter do not distinguish between CSIRTs and SOCs—and we use the CSIRT label to refer to both entities.

Figure 2 is intended to parallel Fig. 1. To avoid redundancies with our previous discussion of end-users, we focus our discussion in this section solely on the unique organizational science opportunities associated with CSIRT work. This future research agenda, which we summarize in Table 3, is driven by two major differences between Figs. 1 and 2.

**Table 3:** Sample Future Research Needs at the Intersection of Organizational Science and Cybersecurity for *Cybersecurity-Focused Employees*

Broad Area for Future Research	Specific Suggestion for Future Research
Behavior or Performance	The availability of numerous metrics or Key Performance Indicators (KPIs) automatically collected through technological means provides numerous avenues for future research on job performance. See the body of the paper for several examples. Cybersecurity-focused employees operate in a constantly changing environment. These employees therefore provide an excellent sample for further research on adaptive performance (Pulakos et al., 2002) and dynamic performance appraisal (Lee & Dalal, 2011; Reb & Cropanzano, 2007). The latter area, in particular, has been characterized by studies of “paper people” (i.e., scenario studies) due to difficulties in obtaining actual time-series objective performance data over a sufficiently long period.
Individual Difference and Team Composition Inputs	Cybersecurity research suggests the importance of, and provides a testbed for, further studying curiosity and resilience as individual differences in challenging, novel, and stressful environments.
Technological Inputs	Trust in automated technology and a view of automated technology as a teammate, topics important in cybersecurity, are topics that have been studied in disciplines adjacent to organizational science (e.g., human factors psychology and engineering), even though these disciplines have built on pioneering work (e.g., the conceptual foundation for trust) in organizational science. Given the proliferation of technology in a variety of work settings, such topics are likely to continue to increase in importance, and organizational science should embrace their study—for instance, by studying human-technology fit using polynomial regression and response surface approaches originally developed for the study of person-environment fit (Shanock et al., 2010).
Team Processes	Cybersecurity frameworks pertaining to the “maturity” of Cybersecurity Incident Response Teams (CSIRTs) focus primarily on technological factors but can be expanded to emphasize “social maturity.” The study of CSIRTs in cybersecurity suggests potentially important gaps in the organizational science research on teams, given that the organizational science literature generally assumes the existence of a standing team. Specifically, the organizational science literature has not yet emphasized “collaboration triggering,” the process by which a single employee, upon examination of a particular problem, determines whether, when, and how to bring in other employees to collaborate on solving the problem. A potentially particularly interesting form of collaboration triggering for organizational science involves “escalation,” in which a lower-level employee brings in a higher-level employee for consultation (Dalal et al., 2016), and which in some ways is the opposite of delegation. The cybersecurity domain suggests the importance of collaboration triggering and in particular escalation, and provides a good test-bed for their study by organizational scientists.
Team-Level Situation	Due to their frequency and their variability in severity, cybersecurity incidents and events (see Table 1 for a distinction) experienced by individual analysts and teams of analysts provide a good test-bed for organizational science theories involving discrete (vs. chronic) situations—for instance, Event System Theory (Morgeson et al., 2015). Given the diversity of potential stakeholders, cybersecurity provides a good test-bed for the study of multiteam systems (MTSs). Although organizational science can help inform the domain of cybersecurity about the influence of diversity on collective problem-solving (e.g., the role of social acuity and the operationalization and effects of diversity), the characteristics of cyber incident response work can generate new insights into how to conduct such problem-solving in contexts that require both high interaction levels and resolution speed.

The first major difference involves the fact that CSIRT employees work in teams and multiteam systems (MTSs). As a result, Fig. 2 is *multilevel*, focusing on the nesting of individual employees within teams and MTSs. Future research would ideally emphasize the dual focus on within- and between-team processes as well as emergent states at the component team and MTS levels. For example, it has previously been theorized in the MTS literature (Zaccaro, Fletcher, & DeChurch, 2017) that there is a high likelihood of countervailing forces within an MTS, such that factors that improve component team functioning may on occasion impair MTS functioning, and vice versa.

Particular contexts of MTSs might compound or magnify these countervailing forces. Descriptions of MTSs distinguish between internal and external forms (Mathieu, Marks, & Zaccaro, 2001). In internal MTSs, all component teams come from the same organization. Accordingly, they will likely possess similar cybersecurity and threat mitigation protocols. However, many cybersecurity incidents require inter-organization collaboration by firms and government agencies. Teams across these organizations form an external or cross-boundary MTS (Zaccaro, Marks, & DeChurch, 2012). This may occur for several reasons: for instance, the interconnectedness of cyber risks in a supply chain (for a review, see Ghadge, Weiβ, Caldwell, & Wilding, 2020), the deployment of employees from one organization to another during significant incidents (e.g., during cyber incidents, the U.S. Department of Energy’s Office of Cybersecurity, Energy Security, and Emergency Response provides emergency support in the form of trained responders who quickly deploy to locations where the electricity sector is being compromised; Sapienza, 2019), or the outsourcing of parts of the cyber MTS (e.g., the forensic analysis team) to other organizations. Such MTSs can often extend across different countries, raising cross-cultural challenges for MTS processes (Tetrick et al., 2016). These contextual aspects of a cybersecurity MTS can impair the information sharing that is crucial for effective response to cyber threat (see also Skopik, Settanni, & Fiedler, 2016). CSIRTs, with their inherent MTS structures operating in a variety of different contexts, provide a useful data source for testing such ideas empirically. Perspectives from research on social networks and inter-organizational ecosystems (Shipilov & Gawer, 2020)—for instance, centrality, complementarities, structural holes, and bottlenecks—could be helpful in addressing such questions.

The second major difference between Figs. 1 and 2 involves the fact that, at the team/MTS level, Fig. 2 represents a CSIRT-specific version of the Input-Process-Output organizing frameworks traditionally used by researchers who study teams—albeit also with a focus on situational and technological factors, as has been recommended recently in the teams literature (Mathieu, Hollenbeck, van Knippenberg, & Ilgen, 2017). As discussed subsequently, we conceptualize process using the rubric of the “social maturity” of the CSIRT. Moreover, in terms of technology, as noted in Fig. 2, an important factor in the context of a CSIRT is Security Information and Event Management (SIEM) software—see Table 1 for a definition—that serves as the first line of defense by generating incoming alerts for human analysts to oversee and handle (Tetrick et al., 2016). Therefore, CSIRTs provide a useful data source for research on teams whose members interact with technology. Moreover, as discussed subsequently, the SIEM collects and in part defines the performance outcomes space in CSIRTs.

### Outcomes

In this section, we focus on *performance* outcomes. However, as noted in Fig. 2, additional outcomes (such as turnover, which is quite high in such occupations; CriticalStart, 2019) can and should also be studied.

Performance outcomes at both the individual analyst and CSIRT (i.e., team/MTS) levels are collected by the SIEM in the form of metrics or, as they are often called, Key Performance Indicators (KPIs). At the CSIRT level, for example, the number of thwarted (vs. successful) attacks is often included among the metrics assessing performance quantity, whereas the percentage of unplanned downtime due to security incidents is often included among the metrics assessing incident handling proficiency (Tetrick et al., 2016).

The availability of these performance metrics suggests numerous avenues for future research that would not only help optimize the functioning of CSIRTs but also address basic research questions associated with the aforementioned criterion problem in organizational science research (Austin & Villanova, 1992). For example, in light of debates regarding better ways to convey effect size information (Brooks, Dalal, & Nolan, 2014) and utility information (Carson, Becker, & Henderson, 1998) to general audiences, how (e.g., display format, level of complexity) should CSIRT performance information and effect sizes ideally be presented to upper management (e.g., the Chief Information Security Officer; see Table 1 for a definition)? As another example, what are the pros and cons of allowing individual employees to monitor their own performance metrics? After all, CSIRT analysts who are permitted to monitor their own “average handle time” might benefit from the near-real-time feedback; however, unless their ability to do so is restricted by design (e.g., via the SIEM) or else controlled for statistically during performance appraisals, they might also attempt to “game” the system by choosing only low-severity incidents that can be resolved quickly. Finally, how can researchers and practitioners best balance the benefits of more information (via metrics) with employees’ desires for privacy and freedom from intrusive performance monitoring (Bhave et al., 2020)?

The availability of these performance metrics is also likely to facilitate the study of organizational science research questions pertaining to performance *change* over time within persons. This is because cybersecurity-focused employees operate in a constantly changing environment—for instance, frequently managing “zero-day” attacks (see Table 1 for a definition). These employees therefore provide an excellent sample for further research on adaptive performance (behavior such as “dealing with uncertain or unpredictable work situations,” “learning new tasks, technologies, and procedures,” and “handling emergencies or crisis situations”; Pulakos, Schmitt, Dorsey, Arad, Borman, & Hedge, 2002, p. 301). As another example, the ready availability of high-volume, high-velocity, and high-variety data—in other words, “big data” (Tonidandel, King, & Cortina, 2018)—involving performance metrics could greatly benefit organizational science research on “dynamic performance appraisal” (Lee & Dalal, 2011; Reb & Cropanzano, 2007), which has thus far been characterized by studies of “paper people” (i.e., scenario studies) due to difficulties in obtaining actual time-series objective performance data over a sufficiently long period.

### Individual difference and team composition inputs

In this section, we focus on individual differences in analysts’ knowledge, skills, and abilities (KSAs), personality, and demographic characteristics. As shown in Fig. 2, when aggregated to the team/MTS (i.e., CSIRT) level, these individual differences are reflected in team composition constructs of interest to organizational researchers: for example, KSA *complementarity*, personality *fit*, and demographic *faultlines*. Other, inherently team-level composition constructs are also of importance: for instance, team size. Here, we focus on two individual differences that were constantly suggested by CSIRT analysts and managers in applied focus groups and interviews (Tetrick et al., 2016; Zaccaro, Hargrove, Chen, Repchick, & McCausland, 2016): curiosity and resilience.

#### Curiosity

Cybersecurity incident response work often requires addressing novel and challenging cyberthreats. This kind of work suggests that successful performance emerges in part from individual differences in curiosity. Curiosity has been defined as “a desire for knowledge that motivates individuals to learn new ideas, eliminate information gaps, and solve intellectual problems” (Litman, 2008, p. 1586). Beyond cybersecurity, curiosity may well contribute to performance in many knowledge work domains similarly characterized by dynamic and novel information environments. However, construct validation and measure development research are still ongoing, such that the dimensionality of curiosity remains unsettled (e.g., Kashdan, Disabato, Goodman, & McKnight, 2020), and the potential utility of the construct in work settings in general, and cybersecurity work settings in particular, is as yet unknown. As regards the workplace, perhaps the best existing evidence is from Mussel (2013), who found that curiosity, measured using the Work-Related Curiosity Scale (Mussel, Spengler, Litman, & Schuler, 2012), explained significant incremental validity beyond 12 other cognitive (e.g., general mental ability, fluid intelligence) and noncognitive (e.g., Big Five personality traits) predictors of job performance. Future research is needed to further examine the operation of curiosity in work environments, such as cybersecurity, that are likely to activate the trait of curiosity.

#### Resilience

4 Cybersecurity incident response is typically stressful and challenging work. Thus, effective performance in such contexts requires individual and collective resilience. Resilience reflects the capacity to maintain high performance in the face of contextual shocks, or to quickly recover previous performance levels following setbacks from contextual shocks (Alliger, Cerasoli, Tannebaum, & Vessey, 2015; Richardson, Neiger, Jensen, & Kumpfer, 1990; Tetrick et al., 2016). Zaccaro, Weis, Hilton, and Jeffries (2011) have defined team or collective resilience as including cognitive resilience (the team’s capacity to focus its collective attentional resources to accomplish effective collaborative problem-solving despite threatening conditions), social resilience (the team’s capacity to maintain its cohesion in the face of threat, as members understand that coordinated action, as opposed to an internal focus on one’s own tasks and needs, is necessary to resolve such threats), and emotional resilience (the team’s capacity to manage emotions both at the individual member and collective levels to avoid destructive emotional contagion in response to high contextual stress).

Although researchers have offered strategies for building social and emotional resilience as well as cognitive resilience (Alliger, et al., 2015; Zaccaro, et al., 2011), more research is needed to apply and validate these strategies in intensive knowledge work environments, such as those that characterize cybersecurity incident response. Additionally, more research is needed to examine resilience within a dynamic stress episode. For example, in a cybersecurity incident, especially a high-severity one, information overload and temporal urgency can cause stress to build. Key questions involve the specific processes—cognitive, social, or emotional—that begin to decay first, the trajectory of perceived stress, and how each type of resilience moderates this trajectory. These are open questions in organizational science in general, and the nature of the prototypical cybersecurity incident response context makes it a particularly fruitful place to examine them.

### Technological inputs

In addition to the “human” inputs discussed previously, the CSIRT consists of technological inputs, often emanating from the SIEM. The SIEM may perform several functions, such as collating cybersecurity notifications from various security technologies into a single location (referred to as log aggregation); providing audit reports to comply with laws, industry regulations, and organizational policies; and performing automated cross-correlation of raw event logs from the network to detect potential incidents (see Table 1 for definitions of the terms “event” and “incident”). At issue, however, is that SIEMs frequently produce false positives (e.g., triggering an alert for a password-guessing application even when multiple failed login attempts are actually due to a user simply forgetting or mistyping his or her password). For instance, it has been estimated that almost half of all CSIRTs experience a SIEM false positive rate of 50% or higher—and that on average up to 25% of a CSIRT analyst’s time is spent chasing false positives (Chickowski, 2019). Moreover, SIEM dashboards vary in user-friendliness and the reports they generate vary in usefulness.

Recent research has expanded the role of technological inputs to include hybrids of human and technological agents interacting to solve team problems. In such arrangements, technology extends beyond the information provision role of SIEMs to more active collaboration. Seeber et al. (2020) note that “Machine teammates could be training for specific collaboration processes, such as coordination, knowledge sharing, or evaluation …which might spark changes in creativity, groupthink, or problem solving” (p. 6). This expansion of technology’s role in teams (i.e., as active teammates) raises a number of very interesting research questions around how to apply insights from the organizational science literature to these kinds of hybrid teams (Poser & Bittner, 2020). To select just two: (1) How do team emergent states such as cohesion, efficacy, and trust form among human and technology-based teammates, and (2) How do humans weigh the contributions of technology in the transition and action phases of hybrid team performance episodes?

A major concern involves the extent to which the CSIRT trusts the SIEM, which is a proximal antecedent to the extent to which the CSIRT relies upon the SIEM. The research literature on trust in automated technological systems is quite relevant here. This is a topic that organizational science researchers have thus far ceded to other disciplines. Human factors researchers in psychology and engineering have taken foundational work on the definition, operationalization, and nomological network of trust from organizational science sources (e.g., Mayer, Davis, & Schoorman, 1995) and have applied it fruitfully to automated systems as the targets of human trust. Important issues examined by human factors researchers in this regard include how trust in automation is (and should be) measured, the antecedents of trust in automation, and the calibration of trust in automation (i.e., the alignment of trust levels with automated system capabilities; Brzowski & Nathan-Roberts, 2019; Schaefer, Chen, Szalma, & Hancock, 2016). Given the proliferation of automated (and artificially intelligent) systems, organizational science should embrace trust in automation as a focal construct of interest. For instance, researchers could fruitfully study human-technology fit using polynomial regression and response surface approaches originally developed for the study of person-environment fit (Shanock, Baran, Gentry, Pattison, & Heggestad, 2010).

### Team processes

Figure 2 makes clear the premise that cybersecurity incident response often carries a strong social load that requires high levels of collaboration. Social load refers to the amount of social capital or resources required for individuals and collectives to solve particular problems (Zaccaro, Weis, Chen, & Matthews, 2014). For example, as problems become more complex in terms of their requisite degrees of interdependence, greater range and diversity of stakeholders, and less familiarity among co-acting individuals and teams, social load increases, along with the necessary expenditure of more social capital (Zaccaro et al., 2014).

Despite this high social load, efforts to improve cybersecurity incident response have focused almost exclusively on technological, structural, and individual-level solutions. In the cybersecurity literature, several frameworks have emerged to define and assess the “maturity” of cybersecurity incident response teams (e.g., Butkovic & Caralli, 2013; NCSC-NL, 2015; Stikvoort, 2010). What is typically missing from such maturity models is the capacity of the team members to collaborate effectively in resolving incidents (Tetrick et al., 2016). Tetrick et al. defined this capacity as the CSIRT’s “social maturity” and identified nine contributing elements: effective communication processes, information sharing, collaborative problem-solving, shared knowledge of members’ and teams’ unique expertise (i.e., transactive memory), trust, collective adaptation, collective learning, conflict management, and effective collaboration triggering norms and processes. Most of these elements are richly represented in the organizational science literature on team effectiveness (e.g., Cannon-Bowers & Bowers, 2011; Mathieu, Gallagher, Domingo, & Klock, 2019). Thus, this is an area where organizational science can provide considerable insight into how to improve the social maturity of cybersecurity teams (see, e.g., Salas, Shuffler, Thayer, Bedwell, & Lazzara, 2015; Steinke et al., 2015).

Conversely, the cybersecurity domain provides an important opportunity for organizational scientists to examine one of the social maturity elements in particular: collaboration triggering (Tetrick et al., 2016). In a cybersecurity context, collaboration triggering refers to the process by which an individual cybersecurity analyst determines whether, when, and how to bring in other analysts or teams of analysts to mitigate an incident as a team or MTS (Tetrick et al., 2016). A specific form of collaboration triggering involves “escalation,” in which a lower-level analyst brings in a higher-level analyst for consultation (Dalal, Bolunmez, Tomassetti, & Sheng, 2016), and which in some ways is the opposite of delegation by a higher-level employee to a lower-level one.

Surprisingly, neither collaboration triggering in general nor escalation in particular has yet received much emphasis in the organizational science literature on teams despite the seeming pervasiveness of these phenomena in organizational settings. Most existing organizational studies instead assume that work is performed either by individuals or by standing teams. Some organizational science studies have examined how leaders make decisions about whether or not to involve collaboration in their decisions (Mls & Otčenášková, 2013; Vroom & Jago, 1988; Vroom & Yetton, 1973)—in other words, how leaders can trigger consultation and collaboration—but similar models have not yet been proposed regarding how individuals who are not leaders can trigger teamwork and/or leadership. The situational and individual difference antecedents of collaboration triggering therefore remain unstudied. Thus, the cybersecurity domain can offer organizational science a context to examine the process of collaboration triggering and to generate propositions that can apply in other organizational contexts (e.g., medical emergency first response).

### Team-level situation

The nature of the team-level situation in CSIRTs (and its effects at both the individual analyst and the CSIRT levels) is poorly understood, and is therefore a fertile area for future research. We focus here on only two illustrative aspects of the situation: the characteristic properties of incidents being attended to by the CSIRT and the types (i.e., diversity) of stakeholders.

#### Incident characteristics

Incidents vary in terms of characteristics such as frequency and severity. Estimates of the number of incidents to which the CSIRT is alerted on any given day vary from fewer than 10 to several hundred (Harsch, 2019; Killcrece, Kossakowski, Ruefle, & Zajicek, 2003; Leune & Tesink, 2006), depending on the year of the estimate (with increases in incident frequency over time), the size of the organization (with larger organizations experiencing more incidents than smaller organizations), the precise definition of the term “incident” (see Table 1 for a distinction between “incident” and “event”), and the operationalization of that definition via the sensitivity of SIEM settings. Incident severity has been defined along multiple dimensions, including the temporal trajectory of damage, the lifecycle stage at which the attack was discovered, the number and status of people who could be affected by the attack, and the potential impact (informational, reputational, and financial) to the organization and to other entities such as organizational clients and the general public (Checklist Incident Priority, n.d.; Cichonski, Millar, Grance, & Scarfone, 2012; Hutchins, Cloppert, & Amin, 2011; Johnson, 2014; Ruefle, van Wyk, & Tosic, 2013). For instance, the costliest cyber attack in history (thus far) was reportedly the 2017 NotPetya malware attack, which was estimated to cost more than $10 billion in damage across several countries—even spreading back to Russia, whose military is believed to have launched the malware in an act of cyberwar targeting Ukraine (Greenberg, 2018). The varied nature of cybersecurity incidents suggests that they would provide a good test-bed for organizational science theories involving discrete (vs. chronic) situations experienced at multiple levels of analysis (e.g., single employee vs. teams of employees)—for instance, Event System Theory (Morgeson, Mitchell, & Liu, 2015).

#### Diversity of stakeholders

As noted earlier, cybersecurity work often carries a high social load. One reason is the larger number of stakeholders that: (a) can be affected by a cyber incident, and (b) may be instrumental in mitigating cyber threats. These can include various units within an organization, such as the ones most affected by a threat, the C-suite team that needs to deal with the strategic and political implications of the attack, and of course the CSIRT itself. Depending upon the severity of the attack, outside stakeholders may include the organization’s external legal team, government and regulatory agencies, partnering organizations, industry-specific guilds, the organization’s clients, and the public at large. The frequently wide scope of impact of a severe incident suggests that its mitigation requires a broad perspective that takes into consideration the social ramifications for different stakeholders. This in turn suggests that cybersecurity leaders will need to employ a range of social acuity skills in their decision making (Zaccaro & Torres, 2020). Smith (1989) argued that, when defining a problem and its requisite solution parameters, “stakeholder identification enables identification of the goals and values to be considered in the problem’s solution” (p. 973). Organizational science research on social problem-solving and the role of social acuity (e.g., Mitchell, Agle, and Wood, 1997; Smith, 1989; Zaccaro & Torres, 2020) can inform this aspect of cybersecurity incident response.

The complexity of more severe cyberattacks suggests that their resolution will require higher levels of interdependence and interactions among diverse stakeholders. In teams, this may mean diversity in terms of functional expertise. Organizational scientists have pointed to a range of challenges linked to this and other forms of diversity in teams, and have offered models of how diversity may relate to team emergent states and performance (Harrison, Price, & Bell, 1998; Harrison, Price, Garvin, & Florey, 2002). These models can provide some clarity regarding the role of diversity in cybersecurity incident response teams. One challenge in applying these models will be the temporal urgency of many cyber incidents. Research on temporal urgency in teams suggests that decision processes become more centralized with less time devoted to problem discussion and exploration of different solution paths (Argote, Turner & Fichman, 1989; Gladstein & Reilly, 1985). However, the nature of cybersecurity incident response as collective knowledge work (Zaccaro, et al., 2016) among analysts with unique expertise suggests that such centralization and limiting of diverse perspectives will impair incident response. Indeed, Tetrick et al. (2016) suggested that, at higher incident severity, interaction between analysts became more frequent and intense, despite the presence of temporal urgency. Thus, although organizational science can help inform the domain of cybersecurity about the influence of diversity on collective problem-solving, the characteristics of cyber incident response work can generate new insights into how to conduct such problem-solving in organizational contexts that require both high interaction levels and quick resolution.

## Conclusion

In our view, a good way to conclude the current manuscript involves identifying potential objections, on the part of the organizational science community, to the ideas presented herein. Accordingly, in Table 4 we present a list of potential objections along with our responses to them. These objections range from the philosophical (e.g., whether we are merely advocating that organizational scientists should chase the newest shiny object) to the level of contribution (e.g., whether we are advocating anything beyond simply applying organizational science knowledge to yet another very specific domain). Perhaps the most important objection to this manuscript, and one with which we certainly concur, involves the underrepresentation of theories and methods associated with macro-organizational science perspectives such as organizational communication and organizational sociology. It is our hope that the objections and response listed in Table 4 further clarify the contributions of this manuscript as well as suggest additional avenues (beyond those in Tables 2 and 3) for future research.

**Table 4:** Potential Objections to Research at the Intersection of Organizational Science and Cybersecurity, and Responses to These Objections

Potential Objection	Response to Objection
*Philosophical Objection #1:* Organizational science has been accused, with justification, of being a “handmaiden” or “cabana boy” to upper management—in other words, a “servant to power.” In our outreach to cybersecurity, there is a high risk that we will repeat previous mistakes.	We agree with this concern. Specifically, vis-à-vis cybersecurity, there is a risk that organizational science research will emphasize the security of the organization over that of the employee. In so doing, there is a second risk that the privacy of the employee will actively be compromised (e.g., through wanton electronic performance monitoring). Finally, there is a risk of over-stigmatizing some of the individuals who compromise organizational cybersecurity (e.g., Edward Snowden and Reality Winner engaged in “insider threat” behavior, to be sure, but they could also be regarded as whistleblowers). We think it is important for authors to be aware of these concerns and for editors and reviewers to raise these concerns early and often during the review process. With that said, we would also note that, for research aiming to understand and increase secure cyber behavior on the part of the end-user, there is some (though not perfect) overlap between benefit to the organization and benefit to the individual employee. For example, training aimed at improving cybersecurity skills and “hygiene” in organizational settings may well exhibit positive spillover effects in providing employees with the skillset to protect their own personal (non-work) data. In general, we call for future research on the extent to which organizational interests and the interests of individual employees coincide versus conflict in the cybersecurity arena.
*Philosophical Objection #2:* Are you not just advocating for organizational science to move on to the newest “hot”/“sexy” topic? Should we, as scientists, not instead be focusing on what is important?	Arguably, organizational science has *always* been hostage to fad, fashion, and folderol (see Dunnette, 1966); so, why stop now? More seriously, organizational science is often argued to exemplify the scientist-practitioner model; therefore, in our view, even members of the field who consider themselves scientists have some obligation to listen to the concerns of practitioners, policymakers, and the general public. Applied concerns are one (though by no means the only) way of defining what is important. To that end, an issue estimated to cost organizations around $6 trillion worldwide by the year 2021 sounds like a good topic to study! With that said, we are actually arguing for research that not only invokes but also appreciably extends existing organizational science research rather than research that ignores existing research in order to start from scratch in a new (“hot/sexy”) domain. For more details regarding this point, see the “Level of Contribution” objection in this table. Moreover, the changing nature of work has always been an important area of study for organizational science researchers. The technology that promised to enable us to make better decisions turns out to also cause information overload and to be a significant entry point for cyber threats. Beyond financial costs to organizations, these threats are associated with well-being costs to employees and clients/customers. Viewing cybersecurity as merely a “hot/sexy” topic only benefits those committing cyber misbehavior at the expense of organizations, their employees, and their clients/customers. Finally, organizational scientists frequently bemoan the fact that the discipline of organizational science has low external visibility and has not made much of an impact on the public consciousness. Interdisciplinary research, such as that proposed in this manuscript, may help to raise the profile of organizational science. Future research from a philosophy of science perspective could also identify the potential contributions and limits of an organizational science-based approach to cybersecurity.^a^
*Philosophical Objection #3:* Organizational scientists simply do not understand the technical aspects of cybersecurity, so how can we contribute to advancing knowledge in that area?	Some organizational scientists do understand the technical aspects of cybersecurity (e.g., those who started in STEM-related fields of computer science and information systems/technology). But, with that said, we accept the premise that most organizational scientists do not understand the technical aspects of cybersecurity. This is not an insurmountable problem. Our view is that research conducted in the cybersecurity domain should be interdisciplinary. In this view, organizational scientists and researchers with a technical focus simply need to understand enough about each other’s fields to be able to: (1) comprehend the nature of the work and, consequently, the nature of the research problems, and (2) communicate effectively with each other. Why is an organizational science perspective important? For too long, the argument has been made that technical models and solutions were sufficient to advance knowledge and practice in this area. But cybersecurity is about more than just the ones and zeros. End-users in organizations are people, as are cybersecurity-focused employees. People think, feel, and behave (often sub-optimally). Moreover, cybersecurity-focused employees generally work in teams and multiteam systems (CSIRTs/SOCs). Studying people in organizations and how they interact with each other and with technology is certainly within the purview of organizational science.
*Publication Venue Objection:* Even if one accepts that organizational scientists can contribute to cybersecurity research, why should such research be published in organizational science journals rather than in venues outside organizational science, as is currently the case? What is wrong with the current state of affairs? If it ain’t broke, why fix it?	The current state of affairs—in which cybersecurity research is published largely in computer science, information technology, and information science journals—is arguably harmful to organizational science in two related ways. First, it can lead to organizational science researchers continuing to neglect cybersecurity research because of the lack of potential for publication in organizational science journals, and therefore the lack of recognition of such work during promotion and tenure, annual performance appraisals, and so forth. In other words, faced with the choice of publishing cybersecurity-focused research in (say) information science journals or not conducting such research at all, many organizational science researchers might choose the latter option. Although this would arguably be a rational decision from the perspective of the individual researcher, we believe it would be harmful both to the state of knowledge in organizational science and to the external visibility of organizational science. Second, the current state of affairs provides little incentive for researchers from other disciplines to work with us. If the “thought leaders” in our own discipline do not find cybersecurity research worthy of publication, why would researchers in other disciplines find us worthy of collaboration?
*Level of Contribution Objection:* Even if one accepts that cybersecurity research should be published in organizational science journals, why should it be published in “top-tier” organizational science journals? Is the level of contribution likely to be sufficient? Specifically, are you advocating anything beyond simply taking what we already know (in organizational science) and applying it to a new domain (cybersecurity)? That does not sound particularly “scientific” or even interesting.	If we were merely advocating the application of what we already know to the new domain of cybersecurity, it might indeed be harder to make the case that such research should be featured in the pages of top organizational science journals (though, as we note in the body of the manuscript, such research could still be published in other, often non-organizational-science, venues). This is why we have emphasized the importance of using the cybersecurity domain as a vehicle for theory elaboration, theory integration, and/or competitive theory testing. This is why we have also emphasized that cybersecurity can provide opportunities for new conceptual ideas, can challenge habitual assumptions, and can provide unique data sources that permit us to test key questions of interest to basic research. In our view, research along these lines will be of appreciable interest to the top organizational science journals.
*Theoretical Approach Objection:* Is it not the case that cybersecurity research is largely devoid of theory? Are there any other applicable theories besides the good old Technology Acceptance Model?	Actually, there has been an appreciable amount of theory utilization in behavioral cybersecurity. For example, deterrence theory, protection motivation theory, neutralization theory, rational choice theory, and requisite variety, to name just a few, have been used in the domain. As far as purely theory-generating work, most of it is in taxonomic development (i.e., theory of diversity) rather than theories of universals. In sum, we would disagree that cybersecurity research is largely devoid of theory. With that said, it is fair to acknowledge that some of the work in information systems and computer science is written in ways that do not emphasize theory building in the same style as organizational science research. However, the theory is still present in “latent” form in the hypotheses and the choices made as part of the study design. If anything, cybersecurity research is well positioned for integrated theoretical work to be applied and developed, and doing so is supposedly a strength of organizational science research. We encourage future research in this area. Finally, as noted in the body of the paper, the cybersecurity domain may also facilitate tests of and modifications to existing organizational theories.
*Unit of Analysis (and Academic Discipline) Objection:* The manuscript ostensibly represents the intersection of cybersecurity and organizational science. Yet, in terms of the units of analysis, the focus of the manuscript is disproportionately on the individual employee and the team. Unsurprisingly, then, the proposed theories and methods are disproportionately from psychology and micro (or at best meso) organizational behavior. Is there no value in reflecting the diversity of theories and methods in organizational science? For example, organizational sociology and organizational communication may have much to contribute.^a^	We are guilty as charged. The current manuscript represents the areas of expertise of its author team. In that sense, it provides an incomplete perspective on the contributions of organizational science to cybersecurity (and vice versa). We agree completely that macro units of analysis, and consequently macro academic disciplines within organizational science, provide perspectives beneficial to the study of cybersecurity. For instance, the Organizational Communications and Information Systems (OCIS) division of the Academy of Management, along with several other macro divisions, is likely to contribute fruitfully to knowledge in this area. We therefore encourage future cybersecurity research emanating from a wider variety of organizational perspectives.

*Note.* ^a^We thank anonymous reviewers for these suggestions

Our goal for this manuscript as a whole is to motivate and facilitate organizational science contributions to cybersecurity as well as cybersecurity contributions to organizational science. With the ever-changing and increasingly technology-mediated nature of work, cybersecurity is not a passing fad or concern; rather, it is likely to persist and increase for the foreseeable future. Organizational scientists should not let a good crisis go to waste.

## Footnotes

1 Of course, it is also the case that several mundane activities performed by organizational science researchers themselves—participant recruitment (e.g., concern about bots impersonating people on Amazon.com’s Mechanical Turk; Dreyfuss, 2018), videoconferences (e.g., “Zoombombing”; Lorenz, 2020), and email (e.g., phishing attacks; see Table 1 for a definition), among others—are subject to cybersecurity concerns. A future paper could create a taxonomy of such concerns, along with the philosophy of science implications associated with the study of cybersecurity by organizational scientists. We thank an anonymous reviewer for this suggestion.

2 Interestingly (for organizational science researchers), Jensen et al. (2017) suggested that the effectiveness of rule-based training can be enhanced with the addition of mindfulness training.

3 However, in so doing, it is critical to keep in mind concerns regarding employee privacy and trust in management (Bhave et al., 2020; Dalal & Gorab, 2016).

4 Here, we are referring to resilience as a characteristic of cybersecurity-focused *employees* rather than as a characteristic of organizational information communication technology *networks*. For the latter usage, see Table 1.
